# Editorial: Interferon signaling in viral pathogenesis of digestive and respiratory tract

**DOI:** 10.3389/fimmu.2023.1223080

**Published:** 2023-06-14

**Authors:** Pin Wan, Qiaoru Tan, Zhen Luo

**Affiliations:** ^1^ Wuhan Institute of Biomedical Sciences, School of Medicine, Jianghan University, Wuhan, China; ^2^ Laboratory of Ministry of Education for Viral Pathogenesis & Infection Prevention and Control, Institute of Medical Microbiology, Jinan University, Guangzhou, China; ^3^ Foshan Institute of Medical Microbiology, Foshan, China

**Keywords:** interferon signaling, coronavirus, enterovirus, human immunodeficiency virus type 1, antiviral response

Interferons (IFNs) are a type of glycoproteins that are produced and secreted from cells in response to stimuli (1). Viral infection triggers production and secretion of IFNs, whereafter IFNs trigger the activation of Janus kinase (JAK)/signal transducers and activators of transcription (STAT) pathway, leading to the expression of hundreds of IFN-stimulated genes (ISGs) that function as antiviral effectors (2). Interferon signaling pathway plays critical roles in host antiviral immunity. Interferon regulatory factors (IRFs) are indispensable ingredients of antiviral responses that control transcription of ISGs (2). In recent years, more and more studies have been presented to uncover the functions of interferon signaling pathways in the process of viral infections. However, emerging important questions related to how interferon signaling plays roles in viral pathogenesis of the digestive and respiratory tract remain unknown.

In this Research Topic, there are seven research articles recruited and six of them published, focusing on the interferon signaling upon viral infection in the digestive and respiratory tract.

As one kind of coronavirus, severe acute respiratory syndrome coronavirus 2 (SARS-CoV-2) infection causes coronavirus disease 2019 (COVID-19) in the recent years, which is predominantly characterized by respiratory symptoms (3). Cheng et al. followed up and collected blood from COVID-19 patients to uncover the longitudinal dynamics of cellular immune responses. The results showed that activation rate of S/N-specific CD8+ T cells was markedly higher than that of CD4+ T cells in recovered patients. A higher percentage of SARS-CoV-2 S/N-specific T cells were activated in recovered patients compared with healthy controls. They speculated that IRFs might be a highly effective antiviral regulator against coronavirus infection. The antiviral effect of IFN-IRF axis on coronavirus infection was also investigated. Duncan et al. suggested that human coronavirus 229E (hCoV-229E) was moderately sensitive to type I/II Interferon, whereas hCoV-OC43 was not. Both viral infections efficiently induced activated IRF1/3/7 and ISGs, indicating that the antiviral response of infected cells was not completely inhibited during viral infection. IRFs Activation has also been found during SARS-CoV-2 infection. Some experiments showed that IRF1/3 were effectively antiviral against hCoV-OC43 infection, whereas IRF3/7 were significantly inhibitory against hCoV-229E infection.

Respiratory syncytial virus (RSV) mainly invades the respiratory tract and allows diseased cells to fuse, behaving as bad cold symptoms in many aspects (4). Du et al. investigated the role of integrin β4 (ITGB4) during RSV infection and influence of ITGB4 deficiency on airway epithelial anti-RSV responses. The experiments showed that ITGB4 deficiency decreased IFN-λ secretion through the epidermal growth factor receptor (EGFR)/IRF-1 pathway in respiratory epithelial cells, which was involved in the enhancement of early RSV infection and the increase of house dust mite (HDM) sensitivity in later life, thereby weakening the pulmonary antiviral response during RSV infection and leading to aggravation of lung inflammation.

Enterovirus D68 (EV-D68) infection may cause mild to severe respiratory disease in children (5). Yang et al. treatment of EV-D68-infected hosts with pterostilbene (Pte) or its major metabolite pinostilbene (Pin) was found to activate host antiviral immunity. They then further investigated the influences of Pte and Pin on EV-D68 infection of human respiratory cells. It was found that the small molecule inhibitors tested significantly disrupted with EV-D68-mediated cytotoxicity, inhibiting replication of viral RNA, synthesis of proteins, and production of infectious viral particles. This confirms that Pte and Pin have antiviral activity against EV-D68.

Human immunodeficiency virus type 1 (HIV-1) invades host immune cells and destroys immune function of the human body (6). In the acute HIV-1 infection phase, the common manifestations are upper respiratory and digestive tract symptoms, such as fever, cough, nausea, and vomiting (7, 8). Karakoese et al. found the potency of different type I IFN isoforms to specifically stimulate NK and T cell responses during HIV-1 infection. They confirmed that IFN-α14 and IFN-β primarily increase cytotoxic T cell responses and decrease IFN-γ responses during HIV infection. However, activation of chronic hyperimmune is not driven by type I IFN. Sertznig et al. found that HIV-1 infection and induction of associated ISGs were associated with expression of low serine/arginine-rich splicing factor 1 (SRSF1) in peripheral blood mononuclear cells and intestinal lamina propria mononuclear cells during acute/chronic HIV-1 infection. SRSF1 expression was transiently suppressed *in vitro* after treatment with specific IFN-α isoforms in HIV-1-susceptible cell lines and primary monocyte-derived macrophages. The work highlights the role described to date of SRSF1 as an IFN-regulated cellular effector that decisively influences HIV-1 LTR transcription and RNA processing, and leading to the IFN-induced unfavorable environment for HIV-1 replication.

In a word, these papers collected in this Research Topic present an up-to-date summary of the insights into the interferon signaling in viral pathogenesis of the digestive and respiratory tract, which shed light on the basis for improved strategies to prevent and treat digestive and respiratory tract-related diseases resulting from viral pathogenesis.

## Author contributions

PW and QT contributed to the writing of the manuscript. ZL contributed to the reviewing and editing of manuscript. All authors approved the submitted version.
